# Effectiveness of Using Voice Assistants in Learning: A Study at the Time of COVID-19

**DOI:** 10.3390/ijerph17155618

**Published:** 2020-08-04

**Authors:** María Consuelo Sáiz-Manzanares, Raúl Marticorena-Sánchez, Javier Ochoa-Orihuel

**Affiliations:** 1 Departamento de Ciencias de la Salud, Facultad de Ciencias de la Salud, Universidad de Burgos, Research Group DATAHES, Pº Comendadores s/n, 09001 Burgos, Spain; 2 Departamento de Ingeniería Informática, Escuela Politécnica Superior, Universidad de Burgos, Research Group ADMIRABLE, Escuela Politécnica Superior, Avda. de Cantabria s/n, 09006 Burgos, Spain; rmartico@ubu.es (R.M.-S.); joo0003@alu.ubu.es (J.O.-O.)

**Keywords:** advanced learning technologies, intelligent personal assistant, blended learning, COVID-19

## Abstract

The use of advanced learning technologies in a learning management system (LMS) can greatly assist learning processes, especially when used in university environments, as they promote the development of Self-Regulated learning, which increases academic performance and student satisfaction towards personal learning. One of the most innovative resources that an LMS may have is an Intelligent Personal Assistant (IPA). We worked with a sample of 109 third-grade students following Health Sciences degrees. The aims were: (1) to verify whether there will be significant differences in student access to the LMS, depending on use versus non-use of an IPA. (2) To verify whether there will be significant differences in student learning outcomes depending on use versus non-use of an IPA. (3) To verify whether there will be significant differences for student satisfaction with teaching during the COVID-19 pandemic, depending on use versus non-use of an IPA. (4) To analyze student perceptions of the usefulness of an IPA in the LMS. We found greater functionality in access to the LMS and satisfaction with teaching, especially during the health crisis, in the group of students who had used an IPA. However, both the expansion of available information and the usability of the features embedded in an IPA are still challenging issues.

## 1. Introduction

### 1.1. Self-Regulation Learning and Advanced Learning Technologies

The use of advanced learning technologies can be an excellent teaching aid for efficient learning processes, especially when adapted to Self-Regulated learning (SRL). The learner can use various technologies to interpret how to approach the resolution of learning tasks and, according to the needs that are detected, the use of a particular learning technology will help to guide the learner towards successful outcomes [1]. Advanced learning technologies are frequently applied within a Learning Management System (LMS). An LMS has many advantages, among which we can highlight personalized attention to the student [2], which makes it possible to increase motivation [3]. Likewise, an LMS can facilitate individual and group work [4], and the use of different resources such as virtual laboratories, computer graphics, flipped learning, and flipped classroom experiences, virtual workshops, and messaging, among others [5]. The use of advanced learning technologies can also facilitate collaborative work within an LMS, such as the application of the Project-Based learning (PBL) methodology [6,7]. Therefore, it can be used for the analysis of multimodal and multichannel data on SRL provided by LMS environments, in which different resources such as smart tutoring, serious games, multimedia resources, augmented reality resources, and virtual reality are applied. In addition, LMS with additional technologies can be used to record information related to eye tracking, physiological records, facial expressions, and speech analysis, among others [8,9,10,11]. Later, these records can be analyzed with statistical and data-mining techniques, through which the learning path of an individual student or a group of students may be consulted during the resolution of different tasks [12]. In addition, the use of LMS with additional technologies can facilitate the use of SRL in almost real time [8]. The collaborative methodology implemented through LMS with additional technologies will guide student learning processes and provide oriented feedback to processes [13]. This methodology is useful through automated and individualized resources, so that the help each student may need is available at any time [14]. Nevertheless, learning autonomy with an LMS is a related disadvantage, due to the need for continuous supervision of the LMS by the teacher. However, improvements to the usability of LMSs have been advanced in recent research by the introduction of intelligent multi-agents, currently found in many automated chat systems. Based on natural voice assistance, these systems can perform many internal and external actions based on the user search queries. The results show that the proposed system can have a positive impact on both students’ perceptions of the usability of an LMS, and student performance [15].

### 1.2. Advanced Learning Technologies and Intelligent Personal Assistant

The use of an Intelligent Personal Assistant (IPA) to improve learning is an emerging practice that, although not yet widespread, has an important future role. The implementation of IPAs through Voice User Interfaces (VUIs) (see Barcelos et al. [16] for an analysis of the taxonomy of voice assistants) means that these assistants can give immediate and intuitive responses to natural language stimuli, so that the user can develop voice interaction through the computer system. In addition, many of them include the possibility of creating applications at no cost for their development and use, such as the Amazon Echo or the Google Home assistants. The system begins from a stimulus (voice) and gives an answer to the query from the user. The characteristics of these IPAs are their functionality, immediate availability or inductiveness, and the empathy they generate with the user, and some are compatible with the Chatbot format [17]. Recent studies have indicated that IPAs can increase their efficiency, if they include the figure of an avatar as an agent of conversational interaction [12]. In educational contexts, IPAs are incorporated in LMSs, such as Moodle (Modular Object-Oriented Dynamic Learning Environment), specifically for the support of learning among students with some type of educational need [18], such as the visually impaired. The functionality offered by IPAs includes guidance for navigation on the platform or on the web [15], guidance for both reading and writing texts [19], and providing feedback on the tests results, for example, quizzes [18]. IPAs are also incorporated in Moodle modules, one example of which is the “Lesson”. Bearing IPA architecture in mind, it can be a tool to build scripts and learning scenarios [17]. This new IPA functionality is potentially effective in virtual labs and simulated environments, as well as when completing quizzes [20]. The incorporation in Moodle of a module called “voicerec” [21] also recently commenced, although this technology is still in an initial state of development and presents implementation difficulties. Its advantages for the user are that it favors coaching and helps the student to find and to access information that has been tested, filtered, and prepared by the teacher. It can also be used at all times, which favors the personalization of learning and, at the same time, promotes collaborative work (teacher-student, student-teacher, student-student, student-materials, etc.). All of these aspects increase student motivation for learning [22].

Regarding the studies on the usability assessment of IPAs, users have indicated that interfaces must be adapted to the needs of each task [23,24]. IPAs that include holograms are under evaluation as learning aids [25,26]. In summary, the type of IPA and its objectives vary and although they are all implemented using natural language, the technology underlying each one is different [27]. In short, IPAs are increasingly finding their way into educational and health-related environments [28,29]. Their advantages are that they encourage personalization in learning [30,31] and in therapeutic intervention [15]. In addition, they can provide insight into patterns of interaction, on which basis students can be provided with personalized interventions [32,33]. Even so, this technology is very complex and is still in an initial state of development [28]. Research studies therefore have emphasized the need for extensive research in this area [34,35].

### 1.3. The Use of Voice Assistants: Applicability in Prevention of Learning Difficulties

As previously noted, current IPA technology incorporates Machine Learning techniques (deep learning and reinforcement learning) resources based on voice-recognition systems [36]. IPAs provide users with information on coursework, facilitating its planning [37,38]. Specifically, the recent use of this technology in university-learning contexts has been associated with very good results and levels of acceptance, specifically among students with special educational needs (visual, auditory, memory, etc.) [39,40]. Furthermore, computer security resources are also incorporated in IPAs, as users must log in before implementing them [41]. Their inclusion in blended learning university learning environments is also beginning to find acceptance, increasing their functional applications [42,43]. IPAs also generate high levels of student satisfaction, as students can access teaching at the most convenient time and place and can receive personalized feedback [44]. The use of this technology also provides a further channel for teachers and academic leaders to connect with students and to understand their main concerns [45]. IPAs can likewise be used to provide students with information on administrative aspects [45,46] and they are very useful for students with visual [47] and auditory needs [48]. These users particularly value the versatility of access to information searches [49]. However, recent studies have also indicated that each IPA needs to be adapted, in terms of both interface and functionality, to respond to the needs of each user [50,51,52]. This field of study still has a long road to travel down, as users currently value the effectiveness of IPAs at only 60% [53].

In conclusion, the world is increasingly turning digital, which implies an urgent need for a series of changes to teaching methods for the inclusion of learning tools within higher education. Experts are calling for an intelligent university in which technology and pedagogy are implemented in teaching–learning environments [54,55]. These environments may be blended learning, or virtual, yet they are quite unlikely ever to be purely face-to-face again. In particular, the global pandemic caused by COVID-19 has quite suddenly underlined the value of telematic teaching tools, prompting governments and university leaders to urge both teachers and students to make good use of these technologies. Research must therefore be conducted to determine the effectiveness of these different resources such as IPAs in blended learning and e-learning spaces [5,56,57].

Based on the research noted above, the research questions in this study are as follows: (RQ1) to verify whether there will be significant differences in student access to the LMS, depending on use versus non-use of an IPA; (RQ2) to verify whether there will be significant differences in student learning outcomes depending on use versus non-use of an IPA; (RQ3) to verify whether there will be significant differences for student satisfaction with teaching during the COVID-19 pandemic, depending on use versus non-use of an IPA; (RQ4) to contrast students’ perceptions of the usefulness of an LMS that incorporates an IPA.

## 2. Materials and Methods

### 2.1. Participants

The convenience sampling process concluded with a sample of 109 third-grade students in Health Sciences degrees: 61 in Group 1 and 48 in Group 2. The sample included all students studying on the third year of a Health Sciences degree at the University of Burgos. In Table 1, the statistics on the two variables, age and sex, can be consulted in Table 1.

### 2.2. Instruments

*(a) The Scale of learning strategies (ACRAr) by Román and Poggioli* [58]: a widely tested instrument in investigations on learning strategies. It is used to identify 32 strategies at different times of processing information: acquisition information (α = 0.78); encoding information (α = 0.92); recovery information (α = 0.83); and metacognition strategies (α = 0.90). In this study, only the metacognitive strategies scale was used. The indicators of scale validity for the sample were metacognition strategies α = 0.90. ACRAr has been widely validated among secondary education and university students [59].

**Figure 1:**
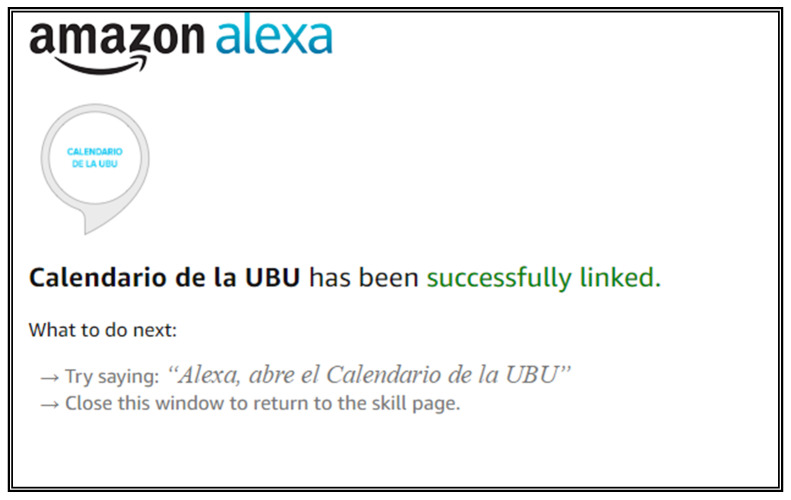
Intelligent personal assistant (IPA). Skill Alexa “UBUVoiceAssistant”.

**Figure 2:**
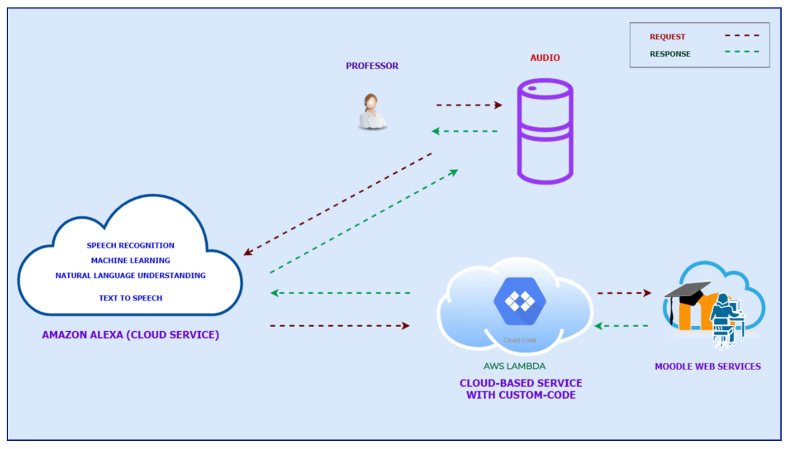
Diagram of the operation of the “UBUVoiceAssistant” application from Moodle.

*(b) Alexa’s Computer application. “UBU(Universidad de Burgos) VoiceAssistant”*: a specific application was developed for students to consult the key dates on the course (delivery of practices, completion of questionnaires, delivery of project, etc.) through a (mobile or computer) device. This application has a client-server within the Alexa service system integrated in the Amazon Web Service (AWS). An example of the interface and operation can be seen in Figure 1 and Figure 2, respectively. Students have first to accredit their identity to enable use of the “UBUVoiceAssistant” computer application. This process is achieved with the use of UBUVirtual, the learning platform (LMS) of the University of Burgos. The students must provide valid credentials in the accreditation of their identity to access the platform. After the successful validation of these credentials, the student is then allowed further access to the “UBUVoiceAssistant” Computer application. The connection is therefore secure, and the protection of personal data is guaranteed [60].

*(c) Scale of assessment of the development of the subject.* The ad hoc development of the scale yielded 18 closed response questions, measured on a 5-point Likert-type scale, and 8 open-ended questions (4 of which refer to the development of teaching during the COVID-19 health crisis) [20]. The total reliability of the scale was α = 0.95 and, for each item, the values were within an interval of α = 0.94–α = 0.96. The scale can be found in the Supplementary Material, Table S1.

*(d) Questionnaire for assessing the functionality of the IPA “UBUVoiceAssistant”.* This instrument consists of two closed-ended questions: a multiple-choice question (with 5 options) a no/yes question, and three open-ended questions. As it is fundamentally a qualitative opinion survey, no reliability analysis could be performed. The questionnaire can be consulted in the Supplementary Material, Table S2.

*(e) Learning Management System “UbuVirtual” based on Moodle 3.7*: UBUVirtual was used in Moodle version 3.7 with a platform design based on a constructivist development designed for personalized learning and collaborative work on the platform [61,62,63].

*(f) eOrientation plugin*: a Moodle plugin, now registered under patent No. BU-09-20, was funded through research project No. BU032G19 awarded for research, in 2019, by the Junta de Castilla y León [64]. The plugin is compatible with Moodle log analysis of student and teacher access to the platform, and interaction with it through the various available activities and resources [65]. This Moodle plugin and its associated graphics can be used to follow the progress of students, for more information see the research of Sáiz-Manzanares, Marticorena-Sánchez, and García-Osorio [65].

*(g) Pedagogical Model*: in both groups, the same pedagogical model was used. The pedagogical model includes the following elements: development and defense of PBL, quiz-type questionnaires, and co-evaluation activities in evaluation processes throughout the teaching–learning process and flipped learning experiences. The effectiveness of this pedagogical model has been tested in various investigative studies [5,6,25,35,56,61,63,65].

### 2.3. Procedure

**Figure 3:**
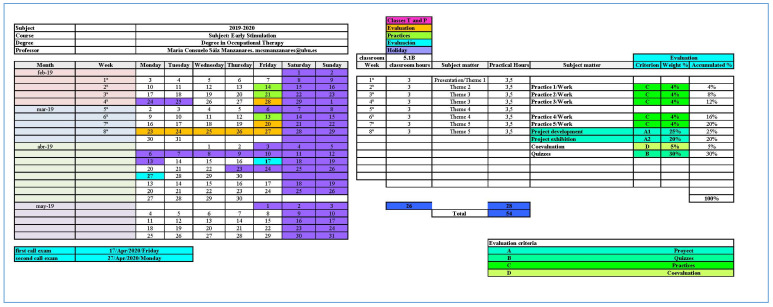
Process and procedure schedule.

Before the study commenced, the authorization of the Bioethics Committee of the University of Burgos and the informed consent of all participants were obtained in writing (see point 2.5). The subject was designed with a blended learning methodology using flipped classroom experiences, which meant that teaching, although delivered in person, was through a Moodle-based LMS (UBUVirtual: learning platform of the University of Burgos), which contained hypermedia resources (videos in flipped classroom experiences and computer graphics). The pedagogical design of the subjects included the following elements: practices (weighted 20% of final grade), quizzes (weighted 30% of final grade), project work, and a defense of a project using practical assumptions drawn from PBL methodology (weighted 25% and 20% of final grade, respectively) and participation in co-evaluation (satisfaction and opinion surveys on the organization of the course) (weighted 5% of final grade). The difference between Group 1 and Group 2 was that, in the second group, an IPA based on the Alexa computer application and integrated into AWS was used from the beginning of the course. Students accessed the IPA using their UBUVirtual credentials. The voice assistant informed the students about events and test deliveries and evaluation procedures in relation to course planning. These events were also collected in a PDF calendar of processes and procedures with assignment dates, available to students from the beginning of the course (an example can be seen in Figure 3). The development of the teaching began on February 3 and ended on April 2 (9 weeks) of 2020. However, on March 12, the Spanish state declared a state of alarm over the COVID-19 health crisis and from that time onwards the teaching was imparted online, exclusively for both groups, over a total period of 4 weeks.

### 2.4. Design and Data Analysis

A quasi-experimental design with an equivalent control and sample group was used for quantitative data analysis. With regard to statistical analyses, the non-parametric Mann–Whitney U test for independent samples was used to check homogeneity between groups before the intervention. Asymmetry and kurtosis analyses were also used to study the normality of the sample. In addition, to check research questions 1, 2 and 3, a single factor fixed-effects ANOVA and the eta-squared formula yielded their respective effect sizes. In addition, a descriptive multidimensional ideographic design was used for the qualitative analysis. The open-ended responses to research questions 3 and 4 were analyzed, first through a categorization of the responses, and then through a frequency and percentage analysis applied to their categorizations. The SPSS v.24 software has been used for data analysis [66].

### 2.5. Ethical Approval

At the beginning of the project, approval was obtained from the Bioethics Committee of the University of Burgos (No. IR 30/2019). The informed written consent of all participants in the study was documented in accordance with the Declaration of Helsinki.

## 3. Results

### 3.1. Previous Statistical Analyses

Before the study began, an analysis of the homogeneity between groups was performed with the ACRAr Metacognitive Strategies Scale [58,59], using the non-parametric Mann–Whitney U test for independent samples, of the responses from the students before the instruction commenced. As can be seen in Appendix A, no significant differences are found in Table A1. Therefore, the groups can be considered similar.

A normality analysis was then performed on the sample distribution on the ACRAr Metacognitive Strategies Scale [58]. Values over |2.00| are indicative of extreme asymmetry and the lower values that the sample follows are indicative of a normal distribution. Kurtosis values between |8| and |20| suggest extreme kurtosis. In this study, as can be seen in Appendix A, Table A2, no extreme values of asymmetry or kurtosis were detected, so it was concluded that the sample followed a normal distribution, and parametric statistics may be applied.

### 3.2. Research Question 1

A fixed-effect factor ANOVA was performed (IPA use vs. non-use) to test RQ1. As can be seen from Table 2, significant differences were found for: the number of accesses to the practice resources on the platform in favor of Group 2, for which IPAs returned a high effect size of 43% [*F*_(1,107)_ = 81.97, *p* = 0.00, η^2^ = 0.43]; access to information on the quiz-tests [*F*_(1,107)_ = 116.25, *p =* 0.00, η^2^ = 0.52] in favor of Group 1 that made no use of an IPA, with a high effect size of 52%; and, access to all information on the platform [*F*_(1,107)_ = 21.81, *p =* 0.00, η^2^ = 0.17] in favor of Group 1 that had made no use of an IPA, with a low effect size of 17%.

### 3.3. Research Question 2

In relation to RQ2, significant differences were only found in favor of Group 1 for the learning outcomes obtained in the practices [*F*_(1,107)_ = 6.02, *p =* 0.02, η^2^ = 0.06] with a very low effect size (Table 3).

### 3.4. Research Question 3

The tests performed on RQ3 may be checked in Appendix A Table A3. The results indicate that the degree of student satisfaction with the development of teaching in which a blended learning methodology was applied was high in both groups (Group 1: *M* = 4.90 out of 5, *SD* = 0.37; Group 2: *M* = 4.90 out of 5, *SD* = 0.34). However, significant differences were found in student perceptions of the following items: item 1 (degree of prior knowledge) [*F*_(1,97)_ = 3.89, *p =* 0.05, η^2^ = 0.04]; item 2 (degree of knowledge after completion of teaching [*F*_(1,97)_ = 4.38, *p =* 0.04, η^2 *=*^ 0.04]: item 3 (clarity of the objectives of the course) [*F*_(1,97)_ = 4.53, *p =* 0.04, η^2 *=*^ 0.50], item 7 (facilitation of group work) [*F*_(1,97)_ = 109.88, *p =* 0.00, η^2 *=*^ 0.54], in this case with a high effect size. All of the results are in favor of the group in which the IPA had been applied. Although significant differences were also found in item 9 (possibilities that the development of the subject offers for future labor market insertion) [*F*_(1,97)_ = 5.35, *p =* 0.02, η^2^ *=* 0.05], in this case in favor of the group in which no IPA had been used.

The open-ended responses on the scale were then analyzed. First, a categorization of the responses given by both groups to the open-ended questions was performed. Secondly, a frequency and percentage analysis by category was applied. Both procedures were performed with the program ATLAS.ti v.8 (see Table A4, from Appendix A). The results indicate that for *question 1* (“Do you think it is convenient to change anything in the subject? Why?”), the highest response percentage was found in Group 2 in the category “There is no need to change anything” (57.89%); in *question 2* (“In your opinion, which units of the current subject should be expanded? In theoretical content or in practical content? Why?”), the highest percentages were found in Group 1 in the category “Nothing” (37.50%) and in Group 2 in the category “Nothing” (54.17%). With respect to *question 3* (“In your opinion, which units of the current curriculum should be reduced? In theoretical content or in practical content? Why?”), the highest percentage was found in Group 2 in the “Nothing” category (70%). With regard to *question 4* (“Please give any indications you consider appropriate for the improvement of the development of the subject”), the highest percentage was found in Group 2 in the category “There is no need to change anything” (66.67%).

Regarding questions on teaching during the COVID-19 state of alert, it was found that in *question 1* (“How has work on the platform been in the weeks following the outbreak of the COVID-19 pandemic alert?”), Group 1 had the highest percentages, in the category “Difficult” (25%) and Group 2 in the category “Very good” (75%); in *question 2* (“After the COVID-19 pandemic alert, the resources of virtual meetings, email and platform support from the teacher have been.”), Group 1 had the highest percentages, in the categories “Increasing the explanations by videoconference” (16.67%) and “Very good” (16.67%) and Group 2 “Very good” (66.67%). In *question 3* (“What would you have added as an aid to teaching during the state of alarm?”), Group 1 had the highest percentage in the categories “Nothing has been taught correctly” (33.3%) and “Nothing, everything has gone very well” (33.3%), and Group 2, in the category “Nothing, this type of methodology has facilitated the continuation of the course” (33.3%). In *question 4* (“Would you include any other resources than those used by the teacher (virtual meetings, email and platform support, etc.) during the COVID-19 pandemic alert?”), Group 1 had the highest percentages in the “Nothing” category (33.33%) and, in Group 2, in the “Nothing” category (66.67%). Regarding *question 5* (What would you have eliminated as a teaching aid during the state of alarm?), Group 1 and Group 2 had the highest percentages, both in the “Nothing” category (50%).

### 3.5. Research Question 4

The responses of the students in Group 2 were analyzed, in order to study RQ4, for which the Scale for Evaluating the Functionality of the IPA “UBUVoiceAssistant” was applied. Questions 1 and 2 were respectively answered, on a Likert-type scale and with a yes/no question. The response rate was 87.75%. Regarding the first closed-ended question (“To consult the events of the subject (dates of delivery of practices, dates of tests type test, etc.), what resource do you use?”), 18.6% used the calendar offered by Moodle on the platform by default, 46.5% consulted the process calendar uploaded by the teacher on the UBUVirtual platform, 14% used the IPA, 11.6% consulted their colleagues and 9.3% had noted the information down since the beginning of the course.

Regarding the second closed-ended question (“Would you like to receive notifications through an IPA, either on your mobile phone or on another platform?”), 81.4% of students opted to continue receiving notifications on the subject and university activities through the IPA.

Answers were categorized for the study of the open-ended questions. Frequency and percentage analyses by category were then carried out on this categorization. All statistical analysis was processed with the ATLAS.ti v.8 tool. With respect to the first open-ended question (“What other information would you be interested in receiving from the UBUVoiceAssistant computer application?”), the answers showed that 10% of the users did not use the IPA, because of the need to open an Amazon account; 20% considered that the application was good, especially for people with special educational needs; 10% never used it; 10% indicated that they would like the application to include notifications when teachers upload resources on the platform; and 50% indicated that they would like the IPA to include information on all subjects during the academic year. Regarding the second open-ended question (“What information would you like the Moodle platform to give you?”), 60% indicated that they would like Moodle to give notices about activities, tests and exam dates. In addition, 40% indicated that they would like Moodle to give them information on resources or activities that the teacher would include in the platform. Regarding the third open-ended question (“If you are not using the UBUVoiceAssistant computer application, please tell us why and make suggestions for improvement”), 90% indicated that they used the IPA, although they would like information on all subjects to be included throughout the academic year. Meanwhile, 10% indicated that they used no IPA, as it is linked to an Amazon account, although they do find this type of application useful.

## 4. Discussion

The results indicate that the total accessing of the platform was lower in Group 1, where no IPA had been used, although the effect size was low. Likewise, more accessing of practical activities and teacher feedback was detected in Group 2, and more accessing of quiz-type activities in Group 1, with a high effect size in both cases (43% to 52%, respectively). With respect to learning outcomes, no better results were found in the group in which IPAs had been used. Likewise, student satisfaction with the development of the teaching was high in both groups, with no differences between either one. However, significant differences were detected for student perceptions of their knowledge, both before and after starting to teach. Differences were also found for student perceptions of the development of group work, which was higher in Group 2. Furthermore, in the qualitative study of the responses, greater satisfaction was found in the group in which IPAs had been applied. Along these lines, although both groups were satisfied with the development of the thematic units, the highest percentage was found in the group in which IPAs had been applied. In addition, the group in which no IPA had been implemented perceived the work during the state of alarm of the COVID-19 health crisis as more difficult than the group in which IPA had been used, a group that perceived the work during this period as very satisfactory. Along these lines, the group that had not implemented IPA indicated that more videoconferencing would have been necessary, and only 16.6% perceived that teaching had been “very good”, compared to 66.67% of the group that had implemented IPAs. In addition, this group explained that the methodology in use had facilitated the smooth development of teaching during this period. Nevertheless, both groups perceived the teaching resources used during the health crisis as adequate, although the percentage satisfaction was always higher in the group in which IPAs were implemented. These results support those found in other research on: the use of advanced learning technologies in the LMS as a good resource for learning regulation [13]; the use of advanced technologies in LMS learning with personalized attention [2,12,14]; the use of PBL in LMS environments for increasing collaborative work [6,7]; and the use of LMS with additional technologies, which, together with a pedagogical model similar to the one applied in this study [15], increased the motivation and the effectiveness of learning among students [3]. In addition, specifically in the group with access to an IPA, greater satisfaction was found with the teaching–learning process [22], with teacher guidance in the teaching–learning process [23,24], and greater general satisfaction [44].

Regarding the assessment of students who had used IPA, it can be seen that the percentage of systematic use was around 14%, 66% of students opted for more visual resources within the Moodle platform (such as the default use of Moodle in the LMS and the calendar of processes and procedures that the teacher has included on the platform), and 20% of students used none at all. In addition, over 80% of students said they would like to receive information on assessment and test delivery processes and procedures through the Moodle platform with an IPA, as well as other information related to cultural events and events related to their area of knowledge at the university. In addition, some fears were expressed that these devices could invade privacy were linked to a reluctance to use IPAs. In summary, students appreciated the possibilities of using IPAs in university settings [45,46,47]; however, they understand that it is a new technology in this field and consider that there are aspects to be improved, both in terms of functionality and interface presentation [48,49,50,51,52,53].

## 5. Conclusions

The results of this study should be treated with caution, because we have worked with convenience sampling that assembled a group of students from the specific knowledge area of Health Sciences. In addition, the results point to the existence of strange variables that may influence the results, such as the learning history of the participants. Future studies will therefore be aimed at increasing both the size of the sample and the knowledge branch of each student, as well as evaluating the student records of collaborative learning. Nevertheless, despite the areas of research improvement, it should be noted that there is very little research that refers to the use of IPAs as a support for university teaching, since their preparation and use in LMS requires a complex technological and fieldwork framework.

The development of teaching in the university context is increasingly justified by the blended learning design and works towards the inclusion of different additional technologies and PBL resources. Within this framework, the pedagogical design of blended learning spaces in LMS is key to the consolidation of the teaching–learning process. Every day, technology offers new resources that can be incorporated into the LMS, including the use of an IPA. Its use is just beginning and requires important technical adjustments, although it is a very promising resource. University teaching has to implement further digitalization and move towards what has been called the smart university. This idea is gaining in strength, and situations such as the COVID-19 health crisis have only accentuated this trend. It is a present need as much a future one, that must be researched from both a technological and a pedagogical perspective, as well as from instructional standpoints. Moreover, both fields have to go hand in hand, since the functionality of technological resources has to be validated in both fields in an interactive manner, reiterating the need for further studies of blended learning. In addition, it is important to consider that the usage of a voice assistant could help students on their learning process, especially during the COVID-19 crisis, with the selected students specifically enrolled on the Health Science degree at the university. We believe that it is important to research about the advanced technological tools available during the current pandemic situation and how those tools can help all Health Science degree students during their learning path, remembering that in our case, the students sample for this study was taken from students within the area of Health Science. As a potential path for future work, we could consider researching how technological aids influence the mood of students studying for degrees who will be directly confronted with situations such as COVID-19.

In summary, this study has contributed innovative results for university learning environments on the use of new technologies: particularly the LMS that incorporates an IPA. Nevertheless, this study has its limitations. As has been indicated, the study has worked with a specific sample size. Future studies will be directed towards expanding the sample, in terms of its size and the heterogeneity of the participating students. Likewise, some qualitative elements have been included in this investigation, although additional elements must be included in future studies, with which triangulation techniques may be applied, to expand the validity of the results. The inclusion of qualitative elements is a great challenge for the advancement of student assessment within virtual university environments. Nevertheless, advancement in this field can only happen with greater investment in both resources and investigation, to confront this challenge with greater assurance.

## 6. Patents

UBUVoiceAssistant Computer application is in the process of being registered [60].

## Figures and Tables

**Table 1:** Description of the sample and the variables: gender and age.

Participant Type			Gender				
	*N*	*n*	Men		*n*	Woman	
			*Mage*	*SDage*		*Mage*	*SDage*
Group 1 (Nursing Degree)	61	5	21.40	0.90	56	23.54	6.30
Group 2 (Occupational Therapy Degree)	48	7	21.71	1.90	41	22.37	2.19
Total	109	12	21.58	1.50	97	23.04	5.01

Note. *Mage* = Mean age; *SDage* = Standard Deviation age.

**Table 2:** A single factor fixed-effects ANOVA (IPA use vs. non-use).

	G1 *N* = 61	G2 *N* = 48	*F*(_1,107_)	*p*	η^2^
	*M (SD)*	*M (SD)*			
Access to practical information	11.48(4.21)	68.85(4.74)	81.97	0.00 *	0.43
Access to information on the quiz-tests	211.48(8.30)	76.71(9.35)	116.25	0.00 *	0.52
Access to project information	30.11(2.72)	30.12(3.07)	0.00	0.99	0.00
Access to co-evaluation information	26.64(2.37)	21.30(2.67)	2.30	0.13	0.02
Access to total information	279.71(11.77)	196.92(13.26)	21.81	0.00 *	0.17

* *p* < 0.05. Note: *N* = number of participants; *M* = Mean; *SD* = Standard Deviation; η^2^ = eta squared (effect size); G1 = Use de IPA; G2 = No use of IPA.

**Table 3:** Single factor fixed-effects ANOVA (IPA use vs. non-use).

	G1 *N* = 60	G2 *N* = 45	*F*_(1,32)_	*p*	η^2^
	*M (SD)*	*M (SD)*			
Learning outcomes in practices	1.99(0.04)	1.96(0.10)	6.02	0.02 *	0.06
Learning outcomes in questionnaires	2.53(0.38)	2.61(0.46)	1.14	0.29	0.01
Learning outcomes in project development	2.17(0.24)	2.14(0.37)	0.35	0.55	0.003
Learning outcomes in defence project	1.82(0.10)	1.85(0.30)	0.60	0.44	0.01
Learning outcomes in co-evaluation	0.19(0.14)	0.19(0.14)	0.01	0.94	0.00
Learning outcomes Total	8.70(0.56)	8.70(1.17)	0.00	0.99	0.00

* *p <* 0.05. Note: *N* = number of participants; *M =* mean; *SD =* standard deviation; η^2^ = eta squared (effect size); G1 = IPA use; G2 = IPA non-use. One participant in Group 1 and 3 participants in Group 2 never completed the course.

## Supplementary Materials

The following are available online at https://www.mdpi.com/1660-4601/17/15/5618/s1, Table S1: Course Development Rating Scale, Table S2: IPA Functionality Rating Scale “UBUVoiceAssistant”.

## Appendix A

**Table A1:** Two independent samples test in parametric test.

Scale of Metacognitive Strategies ACRAr [58]	*U Mann-Whitney*	*p*
1. I am aware of strategies (exploration. underlining. headings) that help me concentrate.	85.5	0.62
2. I am aware of learning strategies that help me to memorize (repetition and mnemonic rules, etc.).	61	0.10
3. I am aware of the strategies that help me to elaborate the information (drawings or graphs. mental images. etc.)	75.5	0.35
4. I am aware of the importance of organizing information by making outlines, sequences, diagrams, maps etc.	95.5	0.98
5. When I need to remember information for an exam. work. etc., I use mnemonic strategies. drawings. concept maps. etc.	92	0.85
6. I am aware that in order to remember information on an exam, it is useful for me to make mental connections with this information.	86	0.63
7. When I prepare an exam, I use strategies to order the information (scripts, diagrams...).	70	0.23
8. I plan the study by selecting the strategies that I think will be most effective.	84.5	0.59
9. Before I answer the questions on a test, I use strategies that help me remember the information.	77	0.36
10. Before I start studying, I distribute the time between all the subjects I have to learn.	92.5	0.87
11. I take note of the tasks I have to perform in each subject.	94	0.93
12. When the exams come up, I make a work plan establishing the time to be devoted to each topic.	87.5	0.70
13. I dedicate a time to each part of the material to study that is proportional to its importance or difficulty.	68	0.19
14. When I study, I check the strategies that work best for me.	93	0.89
15. At the end of an exam, I check the answers recalling the information studied.	87	0.68
16. If the strategies I use to “learn” are not effective, I look for others.	96	1.00
17. I keep the strategies working for me to remember information.	88	0.70

**Table A2:** Analysis of the asymmetry and kurtosis values of the distribution.

Scale of Metacognitive Strategies ACRAr [58]	Range	*Min*	*Max*	*M*	*SD*	*Skewness*		*Kurtosis*	
						*S*	*N*	*S*	*N*
1. I am aware of strategies (exploration. underlining. headings) that help me concentrate.	2.00	2.00	4.00	3.17	0.63	−0.17	0.41	−0.32	0.81
2. I am aware of learning strategies that help me to memorize (repetition and mnemonic rules, etc.).	2.00	2.00	4.00	3.30	0.63	−0.40	0.41	−0.44	0.81
3. I am aware of the strategies that help me to elaborate the information (drawings or graphs. mental images. etc.)	3.00	1.00	4.00	3.17	0.85	−0.70	0.41	−0.29	0.81
4. I am aware of the importance of organizing information by making outlines, sequences, diagrams, maps etc.	2.00	2.00	4.00	3.43	0.66	−0.83	0.41	−0.22	0.81
5. When I need to remember information for an exam. work. etc., I use mnemonic strategies. drawings. concept maps. etc.	3.00	1.00	4.00	3.30	0.77	−1.08	0.41	1.18	0.81
6. I am aware that in order to remember information on an exam, it is useful for me to make mental connections with this information.	3.00	1.00	4.00	3.40	0.65	−1.46	0.41	4.33	0.81
7. When I prepare an exam, I use strategies to order the information (scripts, diagrams...).	3.00	1.00	4.00	3.20	0.86	−1.09	0.41	0.91	0.81
8. I plan the study by selecting the strategies that I think will be most effective.	2.00	2.00	4.00	3.00	0.76	0.00	0.41	−1.22	0.81
9. Before I answer the questions on a test, I use strategies that help me remember the information.	3.00	1.00	4.00	3.13	0.71	−0.81	0.41	1.52	0.81
10. Before I start studying, I distribute the time between all the subjects I have to learn.	3.00	1.00	4.00	3.13	0.83	−0.64	0.41	−0.26	0.81
11. I take note of the tasks I have to perform in each subject.	2.00	2.00	4.00	3.10	0.73	−0.18	0.41	−1.05	0.81
12. When the exams come up, I make a work plan establishing the time to be devoted to each topic.	3.00	1.00	4.00	3.07	0.88	−0.75	0.41	0.08	0.81
13. I dedicate a time to each part of the material to study that is proportional to its importance or difficulty.	3.00	1.00	4.00	2.93	0.84	−0.89	0.41	0.86	0.81
14. When I study, I check the strategies that work best for me.	3.00	1.00	4.00	3.29	0.67	−1.22	0.41	3.29	0.81
15. At the end of an exam, I check the answers recalling the information studied.	3.00	1.00	4.00	3.21	0.89	−1.07	0.41	0.51	0.81
16. If the strategies I use to “learn” are not effective, I look for others.	3.00	1.00	4.00	3.37	0.73	−1.40	0.41	2.50	0.81
17. I keep the strategies working for me to remember information.	2.00	2.00	4.00	3.50	0.42	−1.38	0.41	3.99	0.81

Note. *M* = mean age; *SD* = standard deviation; *A* = asymmetry; *K* = kurtosis; *ASE* = asymmetry standard error; *SEK* = kurtosis standard error.

**Table A3:** Single factor fixed-effects ANOVA (use of IPAs vs. non-use)

	G1 *N* = 57	G2 *N* = 40	*F*_(1,97)_	*p*	η^2^
	*M (SD)*	*M (SD)*			
1. When you started the course your previous knowledge was at one level.	3.86(0.58)	4.05(0.22)	3.89	0.05 *	0.04
2. At the end of the course your knowledge is at one level.	4.00(0.50)	4.20(0.41)	4.38	0.04 *	0.04
3. In your opinion, the objectives of the course have been clear.	4.00(0.53)	4.23(0.48)	4.53	0.04 *	0.05
4. In your opinion, the concepts worked on in the course have been clear.	4.93(0.32)	4.85(0.48)	0.96	0.33	0.01
5. In your opinion, the practices have helped to understand the theoretical concepts.	4.79(0.73)	4.93(0.30)	1.27	0.26	0.01
6. Feedback from the teacher has been quick and accurate.	4.88(0.47)	4.93(0.30)	0.34	0.56	0.00
7. In your opinion, group work has been facilitated.	3.95(0.51)	4.90(0.40)	109.88	0.00 *	0.54
8. In your opinion, all the contents explained in the teaching guide have been addressed.	4.00(0.40)	4.15(0.50)	2.94	0.09	0.03
9. In your opinion, the skills you have developed in this subject can increase your chances of finding work.	4.89(0.40)	4.68(0.50)	5.35	0.02 *	0.05
10. The expectations you had when you enrolled in this course have been met.	4.88(0.50)	4.88(0.40)	0.00	0.98	0.00
11. In your opinion, the procedure and the evaluation criteria were clearly explained	4.88(0.43)	4.83(0.44)	0.34	0.56	0.00
12. In your opinion, the various assessment tests (practical, project-based learning) facilitated learning	4.89(0.45)	4.83(0.45)	0.57	0.45	0.01
13. In your opinion, the use of UBUVirtual as an online teaching platform has been	4.77(0.50)	4.87(0.45)	0.71	0.40	0.01
14. In your opinion, the use of questionnaires to evaluate the development of each unit has facilitated the understanding of it.	3.98(0.44)	3.80(0.61)	2.93	0.09	0.03
15. In your opinion, the difficulty of the subject is at one level.	4.79(0.60)	4.90(0.30)	1.18	0.28	0.01
16. Your level of satisfaction with the development of the practical activities has been	4.72(0.73)	4.88(0.40)	1.51	0.22	0.02
17. Your level of satisfaction with the development of the subject has been.	4.75(0.69)	4.78(0.53)	0.03	0.87	0.00
18. In your opinion, with respect to the rest of the subjects taken in the degree, you value this subject.	4.90(0.37)	4.90(0.34)	0.00	0.96	0.00

* *p <* 0.05.

**Table A4:** Categorization of the answers to the open questions of the scale. Analysis of percentages and frequencies found with ATLAS.ti v. 8

	Question 1 *n* = 19		Question 2 *n* = 24		Question 3 *n* = 9		Question 4 *n* = 6		Question 1. COVID-19 *n* = 8		Question 2 COVID-19 *n* = 6		Question 3 COVID-19 *n* = 3		Question 4 COVID-19 *n* = 2		Question 5 COVID-19 *n* = 2	
	F	%	F	%	F	%	F	%	F	%	F	%	F	%	F	%	F	%
Group 1: Development has been good	2	10.53	0	0.00	0	0.00	0	0.00	0	0.00	0	0.00	0	0.00	0	0.00	0	0.00
Group 1: Further explanations of the content of the practices	1	5.26	0	0.00	0	0.00	0	0.00	0	0.00	0	0.00	0	0.00	0	0.00	0	0.00
Group 1: Increase practices	1	5.26	0	0.00	0	0.00	0	0.00	0	0.00	0	0.00	0	0.00	0	0.00	0	0.00
Group 1: Many deliveries during state of alarm	1	5.26	0	0.00	0	0.00	0	0.00	0	0.00	0	0.00	0	0.00	0	0.00	0	0.00
Group 1: Not	1	5.26	0	0.00	0	0.00	0	0.00	0	0.00	0	0.00	0	0.00	0	0.00	0	0.00
Group 1: Suitable	1	5.26	0	0.00	0	0.00	0	0.00	0	0.00	0	0.00	0	0.00	0	0.00	0	0.00
Group 1: Difficult	0	0.00	0	0.00	0	0.00	0	0.00	2	25.00	0	0.00	0	0.00	0	0.00	0	0.00
Group 1: I don’t think anything	0	0.00	0	0.00	1	10.00	0	0.00	0	0.00	0	0.00	0	0.00	0	0.00	0	0.00
Group 1: Increasing the explanations by videoconference	0	0.00	0	0.00	0	0.00	0	0.00	0	0.00	1	16.67	0	0.00	0	0.00	0	0.00
Group 1: Nothing	0	0.00	9	37.50	1	10.00	0	0.00	0	0.00	0	0.00	0	0.00	1	33.33	1	50.00
Group 1: Nothing has gone very well	0	0.00	0	0.00	1	10.00	0	0.00	0	0.00	0	0.00	0	0.00	0	0.00	0	0.00
Group 1: The development has been very good	0	0.00	0	0.00	0	0.00	1	16.67	0	0.00	0	0.00	0	0.00	0	0.00	0	0.00
Group 1: Very good	0	0.00	0	0.00	0	0.00	0	0.00	0	0.00	1	16.67	0	0.00	0	0.00	0	0.00
Group 1: Nothing has been taught correctly	0	0.00	0	0.00	0	0.00	0	0.00	0	0.00	0	0.00	1	33.33	0	0.00	0	0.00
Group 1: Nothing has gone very well	0	0.00	0	0.00	0	0.00	0	0.00	0	0.00	0	0.00	1	33.33	0	0.00	0	0.00
Group 2: Increasing practical classes	0	0.00	1	4.17	0	0.00	0	0.00	0	0.00	0	0.00	0	0.00	0	0.00	0	0.00
Group 2: Increasing the number of theoretical class	1	5.26	1	4.17	0	0.00	0	0.00	0	0.00	0	0.00	0	0.00	0	0.00	0	0.00
Group 2: Nothing	0	0.00	13	54.17	7	70.00	0	0.00	0	0.00	0	0.00	0	0.00	2	66.67	1	50.00
Group 2: There is no need to change anything	11	57.89	0	0.00	0	0.00	4	66.67	0	0.00	0	0.00	0	0.00	0	0.00	0	0.00
Group 2: To practice in real centres	0	0.00	0	0.00	0	0.00	1	16.67	0	0.00	0	0.00	0	0.00	0	0.00	0	0.00
Group 2: Very good	0	0.00	0	0.00	0	0.00	0	0.00	6	75.00	4	66.67	0	0.00	0	0.00	0	0.00
Group 2: Nothing this type of methodology has facilitated the continuation of the course	0	0.00	0	0.00	0	0.00	0	0.00	0	0.00	0	0.00	1	33.33	0	0.00	0	0.00
Totals	19	100	24	100	10	100	6	100	8	100	6	100	3	100	3	100	2	100

Note. F = frequency; % = percentage.
