# Supplementary material for: Effectiveness of Using Voice Assistants in Learning: A Study at the Time of COVID-19
Source: Int J Environ Res Public Health. 2020 Aug 4;17(15):5618. doi: 10.3390/ijerph17155618 (PMC7432376; doi:10.3390/ijerph17155618)
Supplement: Supplementary file 1 [file ijerph-17-05618-s001.pdf]

International Journal of
Environmental Research and Public Health

MDPI

Supplemental Material

Table S1. Scale of assessment of the development of the subject Sáiz and Marticorena (2018).

Items	Ratings scale				
	**1**	**2**	**3**	**4**	**5**
1. When you started the course your previous knowledge was at one level.	1	2	3	4	5
2. At the end of the course your knowledge is at one level.	1	2	3	4	5
3. In your opinion, the objectives of the course have been clear.	1	2	3	4	5
4. In your opinion, the concepts worked on in the course have been clear.	1	2	3	4	5
5. In your opinion, the practices have helped to understand the theoretical concepts.	1	2	3	4	5
6. Feedback from the teacher was quick and accurate.	1	2	3	4	5
7. In your opinion, group work has been facilitated.	1	2	3	4	5
8. In your opinion, all the contents explained in the teaching guide have been addressed.	1	2	3	4	5
9. In your opinion, the skills you have developed in this subject can increase your chances of finding work.	1	2	3	4	5
10. The expectations you had when you enrolled in this course have been met.	1	2	3	4	5
11. In your opinion, the procedure and the evaluation criteria were clearly explained	1	2	3	4	5
12. In your opinion the various assessment tests (practical, project-based learning) facilitated learning	1	2	3	4	5
13. In your opinion, the use of UBUVirtual as an online teaching platform has been	1	2	3	4	5
14. In your opinion, the use of questionnaires to evaluate the development of each unit has facilitated the understanding of it.	1	2	3	4	5
15. In your opinion, the difficulty of the subject is at one level.	1	2	3	4	5
16. Your level of satisfaction with the development of the practical activities has been	1	2	3	4	5
17. Your level of satisfaction with the development of the subject has been.	1	2	3	4	5
18. In your opinion, with respect to the rest of the subjects taken in the degree, you value this subject.	1	2	3	4	5
**Open questions**					
1. Do you think it is convenient to change anything in the subject? Why?"					
2. In your opinion, which units of the current subject should be expanded? In theoretical content or in practical content? Why?					
3. In your opinion, which units of the current curriculum should be reduced? In theoretical content or in practical content? Why?					
4. Please give any indications you consider appropriate for the improvement of the development of the subject.					
**Questions on teaching during the COVID-19 state of alarm**					
1. How has work on the platform been in the weeks following the outbreak of the COVID-19 pandemic alert?					
2. After the COVID-19 pandemic alert, the resources of virtual meetings, email and platform support by the teacher have been.					
3. "What would you have added as an aid to teaching from the state of alarm?					

4. Would you include any other resources than those used by the teacher (virtual meetings, email and platform support, etc.) during the COVID-19 pandemic alert?	
5. What would you have eliminated as a teaching aid from the alarm state?	

Table S2. Questionnaire for assessing the functionality of the IPA "UBUVoiceAssistant" Sáiz, Marticorena and Ochoa (2019).

1. To consult the events of the subject (dates of delivery of practices, dates of tests type test, etc.).

1. I consult the Moodle calendar in the "UBUVoiceAssistant" platform.	2. I consult the calendar in pdf uploaded by the teacher.	3. I consult the dates in the application "UBUVoiceAssistant".	4. I ask my fellow students.	5. I've had it written down since the beginning of the course with the information given by the teacher in the first class.

2. Would you like to receive notifications via an Alexa device, either on your mobile phone or on another platform?

Yes		No	

3. What other information would you like the Moodle platform to give you?

4. If you are not using the "UBUVoiceAssistant" application, please tell us why and make suggestions for improvement.

© 2020 by the authors. Submitted for possible open access publication under the terms and conditions of the Creative Commons Attribution (CC BY) license (http://creativecommons.org/licenses/by/4.0/).
